# Dentigerous Cyst

**Published:** 1882-02

**Authors:** Eugene S. Talbot

**Affiliations:** Lecturer on Diseases of the Mouth and Jaw, at Central Free Dispensary, Rush Medical College; Chicago


### Clinical Reports.

ARTICLE VI.

DENTIGEROUS CYST. By EUGENE S. TALBOT, M.D., D.D.S., Lecturer on Diseases of the Mouth and Jaw, at Central Free Dispensary, Rush Medical College.

GENTLEMEN :—This patient, David T——, comes to us from Wisconsin for treatment. He is a farmer, twenty-eight years of age ; has always been healthy. About five years ago, he noticed pain and swelling at the septum of the nose, and at the apex of the roots of the central incisors. The teeth are quite loose, and tender to the touch. The tumor has continued to grow until now the face is quite disfigured. You observe the lip is distended to such an extent that the teeth and gums are quite visible. Upon raising the lip, you will readily see the large, dark-colored tumor, extending from the canine on the right to the canine on the left side. By placing my fingers on either side of the tumor and gently tapping it, the movement of fluid is apparent at the opposite finger. I then press upon the hard palate with one finger, which forces the fluid against the finger under the lip ; which proves it to be a sac containing fluid, and that absorption of the anterior portion of the maxillary bones and palate bones had taken place. Is this an alveolar abscess? Let us see. Upon examining the teeth carefully, I find no cavities, and by reflecting the light upon them with a mouth mirror, I discover no discoloration. These facts, together with the slow growth of the tumor, prove that it is not an alveolar abscess. We pass the exploring needle into it to ascertain the kind of fluid it contains, which is safe when there is no doubt about the character of a tumor. We see, upon the removal of the needle, a thick, yellowish, serous fluid at the opening. With the bistoury, I make free incision from one side to the other, and discharge its contents. Upon raising the lip and reflecting the light into it, we see a white, glistening body at the floor of the cavity. With forceps I remove a supernumerary tooth, which will be passed around for examination. We frequently find one of the permanent teeth imbedded in the jaw, causing tumors of different varieties, which are easily diagnosed, from the fact of its absence from its natural position; but a dentigerous cyst caused by a supernumerary tooth is very rare.

---

THE ALIENIST AND NEUROLOGIST, edited and published by Dr. C. H. Hughes, of St. Louis, has completed its second volume, and has thus far fully verified the early promise of its publisher to render practical, for the benefit of the general practitioner, the interesting, widening and instructive departments of psychiatry and neurology.

We are advised by the publisher that the journal is financially on a secure footing, it having been well sustained thus far, and that it will begin its third year with a better table of contents than any hitherto offered, and with flattering professional encouragement and substantial financial aid. The third volume begins January 1, 1882. Subscription, $5.00 per year. The trial of Guiteau brings forcibly to mind the necessity of a discriminating knowledge of psychiatry such as the *Alienist and Neurologist* endeavors to impart.

---

OL. SANT. FLAV.—This has been employed largely for the last twenty years in the treatment of gonorrhœa, and urethral and vaginal discharges generally, says R. Park, M.D., in *The Practitioner*, and to stop a discharge he believes that it is the most specific drug, but it should be continued a fortnight after the latter has stopped. It is efficient in both acute and chronic cases. It certainly has a drying effect upon all the mucous surfaces, when healthy or diseased.

